# Fatty Acids, Amphiregulin Production, and Lung Function in a Cohort of Midwestern Veterans

**DOI:** 10.3389/fresc.2022.773835

**Published:** 2022-03-04

**Authors:** Corrine Hanson, Jana Ponce, Mia Isaak, Art Heires, Tara Nordgren, Chris Wichman, Jeremy D. Furtado, Tricia LeVan, Debra Romberger

**Affiliations:** ^1^Department of Medical Sciences, Division of Medical Nutrition, College of Allied Health Professions, University of Nebraska Medical Center, Omaha, NE, United States; ^2^Department of Internal Medicine, College of Medicine, University of Nebraska Medical Center, Omaha, NE, United States; ^3^VA Nebraska Western Iowa Healthcare System, Omaha, NE, United States; ^4^Colorado State University, Fort Collins, CO, United States; ^5^Department of Biostatistics, College of Public Health, University of Nebraska Medical Center, Omaha, NE, United States; ^6^Harvard T. H. Chan School of Public Health, Boston, MA, United States

**Keywords:** omega 3 fatty acid, DHA, EPA, amphiregulin, lung function, pulmonary rehabilitation, fatty acid (composition), geriatric

## Abstract

**Rationale:**

The relationship between many fatty acids and respiratory outcomes remains unclear, especially with regard to mechanistic actions. Altered regulation of the process of lung repair is a key feature of chronic lung disease and may impact the potential for pulmonary rehabilitation, but underlying mechanisms of lung repair following injury or inflammation are not well-studied. The epidermal growth factor receptor agonist amphiregulin (AREG) has been demonstrated to promote lung repair following occupational dust exposure in animals. Studies suggest the polyunsaturated fatty acid (PUFA) docosahexaenoic acid (DHA) may enhance the production of AREG. The objective of this study was to determine the relationship between fatty acids and lung function in a population of veterans and determine if fatty acid status is associated with concentrations of AREG.

**Materials and Methods:**

Data were collected from a cross-sectional study of veterans within the Nebraska-Western Iowa Health Care System. Whole blood assays were performed to quantify AREG concentrations via a commercially available ELISA kit. Fatty acids from plasma samples from the same patients were measured using gas-liquid chromatography. Intakes of fatty acids were quantified with a validated food frequency questionnaire. Linear regression models were used to determine whether plasma fatty acids or intakes of fatty acids predicted lung function or AREG concentrations. A *p* < 0.05 was considered statistically significant.

**Results:**

Ninety participants were included in this analysis. In fully adjusted models, plasma fatty acids were associated with AREG production, including the PUFA eicosapentaenoic acid (EPA) (β = 0.33, *p* = 0.03) and the monounsaturated fatty acid octadecenoic acid: (β = −0.56, *p* = 0.02). The omega-3 PUFA docosapentaenoic acid (DPA) was positively associated with lung function (β = 0.28, *p* = 0.01; β = 26.5, *p* = 0.05 for FEV_1_/FVC ratio and FEV_1_ % predicted, respectively), as were the omega-6 PUFAs eicosadienoic acid (β = 1.13, *p* < 0.001; β = 91.2, *p* = 0.005 for FEV_1_/FVC ratio and FEV_1_ % predicted, respectively) and docosadienoic acid (β = 0.29, *p* = 0.01 for FEV_1_/FVC ratio). Plasma monounsaturated and saturated fatty acids were inversely associated with lung function.

**Conclusion:**

Opposing anti- and pro-inflammatory properties of different fatty acids may be associated with lung function in this population, in part by regulating AREG induction.

## Introduction

Loss of lung function over time leads to a diagnosis of Chronic Obstructive Pulmonary Disease (COPD), which is a leading cause of death in the United States and the associated social and economic burden continues to grow (1). The International Global Initiative for Chronic Obstructive Lung Disease (GOLD) strategy document summarizes current approaches to COPD management and incudes smoking cessation, influenza vaccination, and pulmonary rehabilitation (1). Nutrition interventions are also recommended in patients who have lung disease and are often included as part of the education provided to COPD patients in pulmonary rehabilitation programs (2). However, there is little guidance or standardization for the type of nutrition education or interventions that would improve outcomes in this patient population (3). Outside of weight maintenance, there are few specific nutrition recommendations that can be provided to patients to target improving respiratory health and tolerance of rehabilitation programs. One randomized control trial reported improved exercise tolerance in individuals with COPD taking omega-3 polyunsaturated fatty acid (PUFA) supplements (4), but the relationship between other fatty acids and respiratory outcomes is not well-studied, and possible mechanisms for the benefits of omega-3 fatty acids are not well-understood.

Ineffective lung repair is considered a key feature of lung function loss and COPD and could impact performance in pulmonary rehabilitation (5). The epidermal growth factor receptor (EGFR) ligand amphiregulin (AREG) has well-established roles in promoting proliferation and repair (6). The regulation of AREG is important; deficient AREG expression can prevent appropriate recovery following injury, but overexpression could lead to fibrotic remodeling. AREG has been found to be increased in the serum of COPD patients and to correlate with measures of lung function decline (7). AREG release is also elevated in damaged airway epithelium in COPD patients (8). AREG is important in the mediation of epithelial proliferation and repair processes, and is also involved in other immune cell activities, including pro-resolution effector cell functions such as T regulatory cell polarization (6, 9). The coordination and appropriate control of these processes is important in the repair and recovery of lung tissue following inflammation and injury.

There are studies to supporting the beneficial role of diets high in omega-3 PUFA in inflammatory lung conditions, including asthma and COPD (4, 10). There are a number of studies demonstrating PUFA-derived lipid mediators are involved in the regulation of inflammation resolution in the lung and promote the production of AREG (11–13). These data identify potential mechanisms for the modulation of lung inflammation and repair by PUFA; however, the mechanistic roles of omega-3 PUFA in the processes of lung repair and protection from lung disease in humans remains unclear, and the impact of dietary intake and plasma concentration of fatty acids on AREG has not been evaluated. The objective of this study was to determine if fatty acid status, as measured by plasma concentrations or intakes of fatty acids, is associated with lung function outcomes or AREG concentrations in a population of subjects at high risk for impaired lung function and COPD.

## Materials and Methods

### Population

This was a secondary analysis of data and samples obtained from a cross-sectional study of agricultural exposures and COPD in veterans seeking health care at the outpatient clinics of the Omaha Veterans Affairs (VA) Medical Center (14). Potential study participants were approached in the primary care outpatient clinics if they had worked on a farm as an adult for >2 years. Eligibility criteria for the study included individuals between the ages of 40 and 80 years. Individuals who had been diagnosed by a physician with asthma, lung cancer or interstitial lung disease such as pulmonary fibrosis, sarcoidosis, and hypersensitivity pneumonitis were excluded from the study. Subject demographics and smoking habits were obtained by in-person and telephone interviews. A participant was considered to be a smoker if they had smoked more than 100 cigarettes in their lifetime. All participants signed a written informed consent document at study enrolment. This study was approved by the VA Nebraska Western Iowa Healthcare Systems Institutional Review Board.

### Lung Function Outcomes

All veterans underwent spirometry and if they had a ratio of forced expiratory volume in 1 second (FEV_1_) to forced vital capacity (FVC) < 0.70, then post-bronchodilator spirometry with 0.083% albuterol was performed. FEV_1_ and forced vital capacity (FVC) were adjusted for height, weight, age, gender, and ethnicity based on NHANES III reference equations (15) for percent-predicted values. Only pre-bronchodilator values were used in this analysis, as not all participants underwent postbronchodilator testing. Outcome variable of interest included: FEV_1_, FEV_1_ percent predicted, FVC, FVC percent predicted, and FEV_1_/FVC ratio.

### Nutrient Intake

The Harvard Food Frequency Questionnaire (FFQ) was mailed to the home address of the participant with a postage-paid return envelope included. Returned FFQs were analyzed by trained personnel in the Harvard University Department of Nutrition. The Harvard FFQ has been validated in adults of all ages and sexes and among a variety of socioeconomic groups (16–21). The FFQ allows for analysis of absolute nutrient intake values from foods and includes intakes from supplements. Specific fatty acid intakes of interest in this analysis were the PUFAs including alpha-linolenic acid (ALA), docosahexaenoic acid (DHA), docosapentaenoic acid (DPA), and eicosapentaenoic acid (EPA). Total omega-3 fatty acid as the sum or DHA+EPA was also considered given the unknown conversion of ALA to DHA. We also analyzed associations with omega-6 fatty acids, including linoleic and arachidonic acid (AA). Based on previous associations with lung function, dietary intakes of individual saturated fatty acids as well as total saturated fatty acids were explored as an additional exposure variable in the analysis (22). Monounsaturated fatty acids were included as an exploratory analysis.

### Plasma Fatty Acid Profile

Fatty acid profile in plasma was determined as previously described by Baylin et al. (23, 24). Briefly, fatty acids are extracted and transmethylated with methanol and sulfuric acid as described by Zock et al. (25, 26). After esterification the fatty acid methyl esters are re-dissolved in iso-octane and quantitated by gas-liquid chromatography as follows: fused silica capillary cis/trans column SP2560, 100 meters X 250 mm internal diameters X.20 mm film (Supelco, Belefonte, PA); splitless injection port at 240°C; hydrogen carrier gas at 1.3 ml/min, constant flow; Hewlett-Packard Model (now Agilent) GC 6890 FID gas chromatograph with 7673 Autosampler injector (Palo Alto, CA); 1 ml of sample injected; temperature program of 90–170°C at 10°C/min, 170°C for 5 min, 170–175°C at 5°C/min, 175–185°C at 2°C/min, 185–190°C at 1°C/min, 190–210 at 5°C/min, 210°C for 5 min, 210–250°C at 5°C/min, 250°C for 10 min. Peak retention times are identified by injecting known standards of purity above 99 percent (NuCheck Prep, Elysium, MN), using Agilent Technologies ChemStation A.08.03 software for analysis. Sample processing and freezing does not affect the fatty acid measurements, as determined by comparison of 2 pools of frozen and fresh samples and short vs. long term freezing. CVs for all the fatty acids studied were monitored continuously by analysis of a pooled control sample (indistinguishable from other study samples) run with each extraction and analysis batch. In general, peaks that are near the sensitivity limit (close to 0.10 of the total area) had larger CVs. Quality control is maintained by external validation through participation in programs offered by both the American Oil Chemists Society and the National Institute of Standards and Technology. Fatty acids quantified in the plasma, as well as their carbon chain name, systematic name, and trivial name are shown in Table 1.

**Table 1:** Fatty acids quantified in serum samples.

	**Carbon name**	**Systematic name**	**Trivial name**
Saturated	8:0	Octanoic acid	Caprylic acid
	10:0	Decanoic acid	Capric acid
	12:0	Dodecanoic acid	Lauric acid, Laurosteanic acid
	13:0	Tridecanoic acid	Tridecylic acid
	14:0	Tetradecanoic acid	Myristic acid
	15:0	Pentadecanoic acid	Pentadecylic acid
	16:0	Hexadecanoic acid	Palmitic acid, Aethalic acid
	17:0	Heptadecanoic acid	Margaric acid, Daturinic acid
	18:0	Octadecanoic acid	Stearic acid
	19:0	Non-adecanoic acid	Non-adecylic acid
	20:0	Eicosanoic acid	Arachidic acid, Icosanoic acid, Arachic acid
	22:0	Docosanoic acid	Behenic acid
	23:0	Tricosanoic acid	Tricosylic acid
	24:0	Tetracosanoic acid	Lignoceric acid
Monounsaturated	14:1n-5c	9c-Tetradecenoic acid	Mysristoleic acid
	15:1n-5c	10c-Pentadecenoic acid	
	16:1n-7c	9c-Hexadecenoic acid	Palmitoleic acid, Zoomaric acid, Physetoleic acid
	18:1n-9c	9c-Octadecenoic acid	Oleic acid, Rapinic acid
	18:1n-7c	11c-Octadecenoic acid	cis-Vaccenic acid
	20:1n-9c	11c-Eicosenoic acid	Gondoic acid
	24:1n-9c	15c-Tetracosenoic acid	Nervonic acid, Selacholeic acid
Polyunsaturated Omega-3	18:3n-3c	9c,12c,15c-Octadecatrienoic acid	Alpha-linolenic acid
	20:5n-3c	5c,8c,11c,14c17c-Eicosapentaenoic acid	EPA, Timnodonic acid
	22:5n-3c	7c,10c,13c,16c,19c-Docosapentaenoic acid	DPA
	22:6n-3c	4c,7c,10c,13c,16c,19c-Docosahexaenoic Acid	DHA, Cervanic aicd
Polyunsaturated Omega-6	18:2n-6cc	9c,12c-Octadecadienoic acid	Linoleic acid, Leinlic acid, Telfairic acid, Linolic acid
	18:3n-6c	6c,9c,12c-Octadecatrienoic acid	Gamma-linolenic acid, Gamolenic acid
	20:2n-6c	11c,14c-Eicosadienoic acid	
	20:3n-6c	8c,11c,14c-Eicosatrienoic acid	Dihomogammalinolenic acid
	20:4n-6c	5c,8c,11c,14c-Eicosatetraenoic acid	Arachidonic acid
	22:2n-6c	13c,16c-Docosadienoic acid	
	22:4n-6c	7c,10c,13c,16c-Docosatetraenoic acid	Adrenic acid

### Dust-Induced AREG

A whole-blood assay was employed to investigate the relationship between plasma fatty acids and amphiregulin release as previously described (27). Briefly, heparinized whole blood was exposed to a well-characterized and potent inflammatory stimulus (1% swine confinement organic dust extract, 1% ODE) for 24 h at 37°C, and AREG was measured in cell-free supernates (ODE-AREG). Dust samples were collected from commercial swine barns and extracts were prepared in an aqueous buffer and sterile filtered as previously described (28). ODE-activated plasma samples were diluted 1:10 in sterile phosphate buffered saline and assayed for soluble human AREG using a commercially available ELISA kit (DuoSet ELISA kit, R&D Systems, Minneapolis, MN, USA) following the manufacturer's instructions. The mean of duplicate measurements for each sample was reported as pg/mL in the undiluted serum. The limit of detectability for the assay was 15.6 pg/mL.

### Statistical Analysis

The association between pulmonary function outcomes (PFO), AREG, and serum and intake dietary biomarkers was examined using linear models. The PFOs modeled are: FEV_1_, FEV_1_ % predicted, FVC, FVC % predicted, FEV_1_/FVC ratio, and natural log of ODE- AREG. The full model considered for each PFO or AREG and biomarker combination included covariates of age, body mass index (BMI), calories, natural log of AREG and smoking status. Backward selection was utilized to arrive at the final model; covariates with a *p* < 0.15 were retained. The covariates sex and race were not considered because >90% of subjects were Caucasian males. Age was considered in PFO % predicted models to examine possible accelerative effects of age. The fit of final models was assessed using residual vs. predicted value, residual quantile-quantile and residual histogram plots. A significance level of 0.05 was used to judge serum and intake biomarkers association with PFOs.

## Results

### Baseline Characteristics

A total of 710 FFQs were mailed out; 101 FFQs were returned for a final response rate of 14%. Ninety-two participants were included in the final analysis, 9 participants were excluded for incomplete exposure or outcome data. The cohort consisted of primarily white males with a mean age of 64.9 years and a mean BMI of 30.8 kg/m2. Mean intake of omega-3 fatty acids (DHA+EPA) was 320 mg/day. Total saturated fatty acid intake was 27.4 g/day, comprising 12% of total energy intake. Baseline characteristics of the respondent population are summarized in Table 2. Mean plasma fatty acid percentages for the cohort are shown in Table 3.

**Table 2:** Demographic characteristics of participating veterans (*n* = 92).

**Variable**	**Mean (SD)**
Age (years)	64.9 (7.5)
Energy intake (calories/day)	1999.2 (890.4)
BMI (kg/m^2^)	30.8 (7.2)
FEV_1_ (L)	2.8 (0.9)
FVC (L)	4.0 (1.0)
FEV1, % predicted	79.9 (23.3)
FVC, % predicted	86.3 (18.7)
Total omega-3 fatty acid intake (mg/day, DHA+EPA)	320
Total saturated fatty acid intake (gm/day)	27.4
α-linolenic acid (gm/day)	1.78
DHA intake (mg/day)	180
EPA intake (mg/day)	140
DPA intake (mg/day)	30
Linoleic acid (gm/day)	14.7
	***N*** **(%)**
Male gender	89 (96.7)
White race	90 (97.8)
**Smoking status**	
Current	14(15.2)
Former	59 (64.1)
Never	19 (20.7)
**COPD**	
Yes	11 (11.9)
No	81 (88.1)

**Table 3:** Fatty acid percent of total fatty acids in plasma of participating veterans (normal ranges not available).

**(Fatty acid)**	**(ug/L mean (SD)**
Octanoic acid	ND^*^
Decanoic acid	ND^*^
Dodecanoic acid	0.02 (0.04)
Tridecanoic acid	ND^*^
Tetradecanoic acid	0.43 (0.55)
Pentadecanoic acid	0.11 (0.05)
Hexadecanoic acid	17.75 (3.17)
Heptadecanoic acid	0.32 (0.06)
Octadecanoic acid	9.54 (1.94)
Non-adecanoic acid	0.03 (0.01)
Eicosanoic acid	0.20 (0.05)
Docosanoic acid	0.45 (0.13)
Tricosanoic acid	0.19 (0.05)
Tetracosanoic acid	0.35 (0.11)
9c-Tetradecenoic acid	0.01 (0.02)
10c-Pentadecenoic acid	0.00 (0.01)
9c-Hexadecenoic acid	1.79 (0.95)
9c-Octadecenoic acid	21.34 (2.74)
11c-Octadecenoic acid	1.78 (0.26)
11c-Eicosenoic acid	0.20 (0.05)
15c-Tetracosenoic acid	0.49 (0.17)
9c,12c,15c-Octadecatrienoic acid (alpha-linoleic acid)	0.63 (0.22)
5c,8c,11c,14c17c-Eicosapentaenoic acid (EPA)	0.69 (0.42)
7c,10c,13c,16c,19c-Docosapentaenoic acid (DPA)	0.56 (0.17)
4c,7c,10c,13c,16c,19c-Docosahexaenoic acid (DHA)	1.43 (0.62)
9c,12c-Octadecadienoic acid (Linoleic acid)	28.14 (3.88)
6c,9c,12c-Octadecatrienoic acid	0.49 (0.18)
11c,14c-Eicosadienoic acid	0.30 (0.07)
8c,11c,14c-Eicosatrienoic acid	1.63 (0.36)
5c,8c,11c,14c-Eicosatetraenoic acid	8.05 (2.31)
13c,16c-Docosadienoic acid	0.01 (0.01)
7c,10c,13c,16c-Docosatetraenoic acid	0.37 (0.14)

* *Not detectable in plasma samples of participants*.

### Fatty Acids and ODE-AREG Concentrations

In the fully adjusted models, two plasma fatty acids showed significant and opposing associations with concentrations of ODE-AREG. These included the PUFA EPA: (β = 0.33, *p* = 0.03) and the monounsaturated fatty acid octadecenoic acid: (β = −0.56, *p* = 0.02). There were no significant associations between intakes of fatty acids and plasma AREG concentrations.

### Fatty Acid Intakes and Lung Function

The only association between intake of any fatty acid and any outcome variable was between total saturated fatty acid intake and FEV_1_/FVC ratio. In the fully adjusted model, the association remained significant with a β-coefficient of 0.002; *p* = 0.04, indicating that for every 1 unit (gram) increase in intake saturated fat, the FEV1/FVC ratio increases by 0.002.

### Plasma Fatty Acids and Lung Function

Multiple associations between plasma fatty acid concentrations and lung function outcomes were present in the fully adjusted models. The omega-3 PUFA DPA was positively associated with lung function (β = 0.28, *p* = 0.01; β = 26.5, *p* = 0.05 for FEV_1_/FVC ratio and FEV_1_ % predicted, respectively), as were the omega-6 PUFAs eicosadienoic acid (EDA) (β = 1.13, *p* < 0.001; β = 91.2, *p* = 0.005; and β = 2.83, *p* < 0.02 for FEV_1_/FVC ratio,FEV_1_ % predicted, and FEV_1_, respectively) and docosadienoic acid (β = 0.29, *p* = 0.01 for FEV_1_/FVC ratio) (Table 4). Five different saturated fatty acids showed significant associations with lung function outcomes, with 4 out of 5 indicating an inverse relationship (Table 4). Similar to saturated fatty acids, monounsaturated fatty acids demonstrated inverse relationships with lung function, with 3 of the 4 significant associations showing negative β-coefficient values. Effect sizes and significance levels for all associations are given in Table 4. There were no significant associations between plasma fatty acids and FVC % predicted.

**Table 4:** Significant associations between fatty acids and lung function outcomes in fully adjusted models.

		**FEV1/FVC ratio**		**FEV**_**1**_		**FEV**_**1**_ **% predicted**	
	**Serum fatty acid**	***β***	***p***	***β***	***p***	***β***	***p***
Monounsaturated	Mysristoleic acid	−4.71	<0.001	−14.05	0.005	−372.7	0.006
	Pentadecenoic acid	−13.75	<0.001	−34.6	0.02	−921.6	0.01
	Octadecenoic acid	−0.15	0.01	-	-	-	-
	Tetracosenoic acid	-	-	3.42	0.04	-	-
Polyunsaturated Omega-6	Eicosadienoic acid	1.13	<0.001	2.83	0.02	91.2	0.005
	Docosadienoic acid	-	-	−15.93	0.02	−491.8	0.007
	Docosatetraenoic acid	0.29	0.01	-	-	-	-
Polyunsaturated Omega-3	Docosapentaenoic acid	0.28	0.01	-	-	26.5	0.05
Saturated	Dodecanoic acid	−1.00	0.02	-	-	-	-
	Tridecanoic acid	−0.12	<0.001	-	-	−10.17	0.01
	Pentadecanoic acid	−1.07	0.003	-	-	-	-
	Hexadecanoic acid	−0.02	0.0004	-	-	-	-
	Heptadecanoic acid	-	-	3.03	0.02	-	-
	9c-Octadecanoic acid	0.052	<0.001	0.11	0.01	3.59	0.002

### AREG (as a Predictor Variable) and Lung Function

Plasma ODE-AREG concentrations (as a predictor variable) were not associated with any lung function outcomes.

## Discussion

The American Heart Association (29), the Academy of Nutrition and Dietetics (30), and in the 2015–2020 Dietary Guidelines for Americans (31) recommend regular intake of fish/seafood in the general population, providing about 250–500 mg/day of EPA and DHA to promote health and reduce the risk of cardiovascular disease. The population of veterans in this study showed intakes of omega-3 PUFAs that are within current guidelines. Analysis of surveys such as the National Health and Nutrition Examination Survey (NHANES), which is designed to assess the health and nutritional status of adults and children in the US, have revealed intakes of EPA and DHA that are well-below recommended levels (32–34). Analysis of the 2003–2008 NHANES cycles found that in US adults, the mean intake of EPA and DHA from foods was 20 and 60 mg/day, and 40 and 70 mg/day when accounting for foods plus supplements (33). Another study which used NHANES 2003–2008 cycles and included potential conversion of alpha-linolenic acid and stearidonic acid to DHA and EPA reported that the mean total omega-3 fatty acid intake was 170 mg/day, and over 90% of the study population (*n* = 24,621) consumed less than the recommended ~500 mg/day (35). Another analysis including updated NHANES cycles reported similarly inadequate EPA and DHA intake (32). The main dietary sources of omega-3 fatty acids are fish and seafood, contributing up to 71% of total intake (36). Our cohort was from a Midwestern state where fish intake is commonly low, however; the calculations for DHA and EPA intakes in our study included any use of supplements, which may account for the level of intake seen in this cohort.

In this analysis, we did not see a significant association between PUFA intake and lung function. However, other studies have reported beneficial effects of omega-3 fatty acid intake on lung outcomes. A recent systematic review identified 11 observational studies evaluating the relationship between COPD and omega-3 fatty acid status, defined by either intakes or serum levels. Six of these studies identified a significant relationship, with 5 studies demonstrating protective effects. The remaining 5 studies had null findings (37). Patterns of dietary intake that show an increased consumption of fish have been associated with a decreased development of COPD in both smokers and non-smokers, as well as increases in lung function (FEV_1_) and decreased long-term COPD mortality (38–40). Fruit and fish intake together explained about 67% of the variation in COPD mortality rates after 25 years in the Analysis of the Seven Countries Study, a population-based cohort of 12,763 men (41).

This study did find a significant, positive relationship between saturated fat intake and lung function parameters (FEV_1_/FVC ratio). These findings are similar to a 2019 report using National Health and Nutrition Examination Survey (NHANES) data documenting that a lower intake of SFA was associated with reduced measures of lung function in people with COPD (22). After adjustment for relevant confounders, percent predicted FVC (β = −3.3, *p* = 0.04 for quartile 1 vs. quartile 4); FEV_1_ (β = −126.4, *p* = 0.04 for quartile 1 vs. quartile 4), and FVC (β = −165.8, *p* = 0.01 for quartile 1 vs. quartile 4) were specifically associated with intakes of saturated fat (22). This analysis of diet in NHANES included short-chain saturated fatty acids (SCFA, C:4-8), and it was hypothesized that anti-inflammatory properties of some SCFA may have contributed to the beneficial effects seen (42). In contrast to the positive association between saturated fatty acid intakes and FEV_1_/FVC ratio in the NHANES study, plasma concentrations of saturated fatty acids in this study primarily showed inverse associated with lung function outcomes. High amounts of saturated fatty acids have been shown to increase blood levels of low-density lipoproteins (LDL), which is a known biomarker of risk for the development of cardiovascular disease (43). Recent studies also report that saturated fatty acids activate the NF-κB pathway and trigger an inflammatory response, acting as non-microbial TLR4 agonists (44). Saturated fat can also trigger a pro-inflammatory response via upregulating NLRP3 inflammasome activity (45). Mouse models have shown that saturated fat increases lung alveolar macrophages, augmenting inflammation in the airway (46). In a study of asthmatic patients fed a single high-fat meal, there was an increase in circulating total, saturated, and monounsaturated fatty acid levels, which was correlated with increases in sputum neutrophils and TLR4 mRNA expression (47). However, recent studies have shown that risk for chronic disease development varies based on both the carbon chain length of the SFA, or the dietary sources (i.e., milk vs. meat), highlighting the fact that relationships between SFA intake and disease may be more complex than originally thought (48–50). While the FFQ used in this study did have the ability to capture intakes of SCFA, these same SCFAs were not present at high enough concentrations in plasma to be detected. Therefore, the plasma analysis of fatty acids would be skewed toward the longer-chain saturated fatty acids, which have been shown to be detrimental in other disease states, while not capturing any benefits of SCFA.

Several plasma PUFAs showed positive associations with lung function, including the omega-3 fatty acid DPA. DPA has recently emerged as a fatty acid of interest in respiratory research due to recent reports of beneficial effects on lung outcomes. The omega-3 fatty acid DPA was positively associated with FEV_1_, FVC, and FEV_1_/FVC ratio in meta-analyses across seven cohorts (*n* = 16,134), with both sex and smoking modifying the relationship (51). Analysis of fatty acid intakes in the Lovelace Smokers cohort has shown that DPA was associated with a better average FEV_1_, and that the FEV_1_ decline from the adverse effect of continuous smoking was neutralized with high omega-3 DPA intake (52). Similar to EPA and DHA intake, reported intakes of DPA in the US population are low (53). However, while EPA and DHA intake decreased during the 2009–2014 period, DPA intake showed a marked increase, while revealing significant differences between different groups based on age and race/ethnicity (53). In addition to the associations between lung function and omega-3 PUFAS, the omega-6 PUFAs eicosadienoic acid (EDA) and docosatetraenoic acid showed positive associations with lung function, while the omega-6 fatty acid docosadienoic acid showed inverse associations with lung function. Very little is known about the specific actions of docosatetraenoic acid and docosadienoic acid, but one study using animal models demonstrated that EDA was able to reduce the pro-inflammatory effects of other omega-6 fatty acids (54).

This study did find inverse association between plasma monounsaturated fatty acids and lung function. Little is known about the relationship between monounsaturated fatty acids and lung disease. Effects of monounsaturated fatty acids on risk of coronary heart disease (CHD) have been explored in meta-analyses with mixed findings. One meta-analysis reported an increase in CHD events, however, most meta-analyses report a diet high in monounsaturated fatty acids to be protective against CHD (55). This serves to highlight paucity of information regarding lipidomics and lung disease, as well as the complex metabolism and actions of lipids and necessity of avoiding overgeneralizations of “good vs. bad” fats.

Two plasma fatty acids in this study show significant associations with ODE-AREG: plasma concentrations of the omega-3 PUFA EPA, which was positively associated with ODE-AREG; and the monounsaturated fatty acid octadecenoic acid, which was inversely associated with ODE-AREG concentrations. The direction of the associations between these fatty acids and ODE-AREG are similar to the directions seen between plasma fatty acids and lung function. Very little is known about potential relationships between fatty acids and AREG, although preclinical investigations have shown the potential for PUFAs to impact this mechanism. In 2018, Nordgren et al. explored how DHA and epidermal growth factor receptor (EGFR) modulate lung repair processes following ODE-induced injury. In their study, bronchial epithelial cells (BEC) were treated with ODE in the presence of DHA and AREG or EGFR inhibitors. Mice were also exposed to ODE intranasally with or without EGFR inhibition and DHA. *In vitro*, ODE exposure induced AREG release from BEC, and DHA treatment enhanced this release. Both DHA and AREG enhanced the repair capabilities of BEC and rescued ODE-induced recellularization deficits. In mouse models, DHA treatment enhanced the production of AREG following exposure to ODE (56). Taken together, these data could indicate a role for fatty acids in the process of tissue repair after inflammatory lung injury caused by environmental exposures.

## Limitations

Our study has several limitations. Our response rate to the survey was low, and was impacted, in part, by the length of time separating the parent study from this secondary analysis of several years, and many of the original study participants were deceased or did not have a valid address or other contact information. Our population is a Midwestern population with agriculture backgrounds, primarily male and white, which may limit generalizability to other populations. Residual confounding is often a concern in lifestyle studies, and some significant findings were attenuated after adjustment for confounders. Additionally, there are some potential covariates, such as corticosteroid use, for which no adjustment was made. We assessed nutrient intake using FFQ methodology, which is subject to various forms of bias, including recall bias and measurement error in the estimated portion sizes of foods, However, our inclusion of both plasma levels fatty acids and nutrient intake levels provides a robust level of exposure assessment, and our use of serum concentrations of fatty acids represents a biomarker-based approach to strengthen these investigations.

## Conclusion

This study provides hypothesis-generating data regarding relationships between serum concentrations of previously unstudied fatty acids with regard to lung function, and demonstrate that in humans, the opposing anti- and pro-inflammatory properties of different fatty acids require further study.

## Data Availability Statement

The datasets presented in this article are not readily available because restrictions apply to this dataset. Requests to access the datasets should be directed to ckhanson@unmc.edu.

## Ethics Statement

The studies involving human participants were reviewed and approved by VA Nebraska Western Iowa Healthcare System. The patients/participants provided their written informed consent to participate in this study.

## Author Contributions

CH, JP, TN, TL, and DR: conceptualization. CH, MI, AH, TN, CW, JF, TL, and DR: methodology. AH and JF: validation. CW: formal analysis. CH, JP, MI, AH, TN, CW, TL, and DR: investigation. CH, TN, TL, DR, JF, and AH: resources. CH, MI, TL, CW, and DR: data curation. CH, JP, and MI: writing–original draft preparation. CH, JP, MI, AH, TN, CW, JF, TL, and DR: writing–review and editing. CH: supervision. CH, MI, TL, and DR: project administration. CH, TN, TL, and DR: funding acquisition. All authors contributed to the article and approved the submitted version.

## Funding

This study was funded by a grant from the United States Department of Veteran's Affairs Merit Award.

## Conflict of Interest

The authors declare that the research was conducted in the absence of any commercial or financial relationships that could be construed as a potential conflict of interest.

## Publisher's Note

All claims expressed in this article are solely those of the authors and do not necessarily represent those of their affiliated organizations, or those of the publisher, the editors and the reviewers. Any product that may be evaluated in this article, or claim that may be made by its manufacturer, is not guaranteed or endorsed by the publisher.
